# The Psychological Impact of the COVID-19 Epidemic on Guangdong College Students: The Difference Between Seeking and Not Seeking Psychological Help

**DOI:** 10.3389/fpsyg.2020.02231

**Published:** 2020-09-04

**Authors:** Shun-Wei Liang, Rong-Ning Chen, Li-Li Liu, Xue-Guo Li, Jian-Bin Chen, Si-Yao Tang, Jing-Bo Zhao

**Affiliations:** ^1^Department of Psychology, School of Public Health, Southern Medical University, Guangzhou, China; ^2^Mental Health Center, School of Public Health, Southern Medical University, Guangzhou, China

**Keywords:** psychological help-seeking, mental health, COVID-19, college students, fear

## Abstract

**Background:**

Coronavirus disease 2019 (COVID-19) has considerably psychologically impacted Chinese college students. Several types of online mental health services were widely implemented for college students during the outbreak. This study investigated the relationship between college students’ mental health status and psychological help-seeking behavior to test the phases-decision-making model (PDM).

**Methods:**

A cross-sectional survey was conducted among college students in Guangdong Province using an online platform. In total, 4,164 students were assigned to the “counseling group” or “non-counseling group” according to whether they had sought psychological help because of the COVID-19 outbreak; the groups were matched based on age, sex, and grade. Demographics, perceived mental health, and experience with seeking psychological help were recorded. Fear, depression, and trauma were assessed by the COVID-19 Fear Screening Scale, Patient Health Questionnaire, and Impact of Event Scale-6.

**Results:**

The fear, depression, and trauma scores were significantly higher in the counseling group than in the non-counseling group (*P*_s_ < 0.001). Fear (OR = 1.27, *p* < 0.001), depression (OR = 1.02, *p* = 0.032), trauma (OR = 1.08, *p* < 0.001), poor perceived mental health status (OR = 3.61, *p* = 0.001), and experience with seeking psychological help (OR = 7.06, *p* < 0.001) increased the odds of seeking psychological help.

**Conclusion:**

During the COVID-19 epidemic, the rate of psychological help-seeking was still low, and college students in poor psychological condition sought psychological counseling more. Fear, depression, trauma, experience with seeking psychological help, and perceived mental health can effectively predict psychological help-seeking behavior. These findings emphasized the importance of closely monitoring college students’ psychological status, providing psychological intervention, and improving the probability of seeking psychological help.

## Introduction

In December 2019, the coronavirus disease 2019 (COVID-19) epidemic emerged in Wuhan, China, started to spread nationwide, and subsequently attracted worldwide attention. The World Health Organization (WHO) has recently declared the COVID-19 outbreak a Public Health Emergency of International Concern (PHEIC) (World Health Organization [WHO], 2020). As of February 25, 2020, a total of 81,109 laboratory-confirmed cases had been documented globally (Holshue et al., 2020; Phan et al., 2020; Rothe et al., 2020), and 78,064 confirmed pneumonia cases and 2,715 confirmed deaths had been reported in China (Chinese Center for Disease Control and Prevention, 2020). Guangdong Province ranked first behind Hubei Province in the number of confirmed cases, with a total of 1,347 reported in 1,447 counties and districts (Guangdong Provincial Center for Disease Control and Prevention, 2020). The COVID-19 pandemic has led to unprecedented threats to humans’ lives and health.

COVID-19 is an unknown, severe, lethal, and readily transmissible new infectious disease. Massive infectious disease outbreaks usually have a considerable impact on human survival (Chih-Hung et al., 2006), and this COVID-19 epidemic has been no exception. The COVID-19 outbreak has had a profound impact on the daily life of people living in affected areas and on society as a whole. Major sporting events and cultural activities have been canceled, businesses have suffered and closed, the use of public transportation has dramatically decreased, classes have been suspended, and the death toll continues to mount. The implementation of unprecedentedly strict quarantine measures in China has kept a large number of people in isolation (Qiu et al., 2020), and all citizens have been asked to stay at home and go out less. However, the COVID-19 epidemic has not only affected the daily life and physical health of ordinary Chinese but has also caused many psychosocial problems.

Studies have pointed out that the psychological impact of public health emergencies is long-lasting (Chang et al., 2020). The outbreak of COVID-19 can be regarded as a mental health catastrophe. In contrast to common life stressors, the COVID-19 epidemic represents an acute, large-scale, and uncontrollable stressor. Generally, psychosocial responses to such stressors are varied and include feelings of anxiety, shame, personal and social failure, or weakness (Verghese, 2004); underestimation of the possibility of survival; overestimation of the likelihood of infection (Koh et al., 2005); excessive and inappropriate preventive measures; and increased demand for healthcare services in a time of shortage (Rosling and Rosling, 2003). The results of a national survey showed that 98.54% of respondents felt excessive fear, worry, and nervousness, believing that the epidemic posed a serious threat (Chen et al., 2020). Another study pointed out that almost 35% of the general population reported experiencing psychological distress due to the COVID-19 epidemic (Qiu et al., 2020). In addition, an internet-based survey found that public anxiety and panic were relatively high, and ∼32.4% of the respondents were assessed as having depressive symptoms (Cai et al., 2020).

Individuals exposed directly or vicariously to life-threatening situations have a high prevalence of psychological morbidity (Weiss et al., 1995; Catalan et al., 1996). The impact of the COVID-19 outbreak on college students, as a special group in society, cannot be ignored. A study found that individuals between 18 and 30 years of age or above 60 presented the highest scores on the COVID-19 peritraumatic distress index (CPDI) (Qiu et al., 2020). Chinese college students have been exposed to a significant number of COVID-19-related stressful events during the outbreak, including disruptions to their academic, leisure, family, and social life. These disruptions have been shown to frequently cause boredom, frustration, anxiety, fear, and a sense of isolation from the rest of the world (Brooks et al., 2020; Chang et al., 2020), leading to distress among college students.

When psychological distress occurs, asking for psychological help is a way to cope. The National Health Commission of China has published several guideline documents to better address psychological problems in the Chinese population during the COVID-19 epidemic period. The rapid transmission of the virus between people impedes traditional face-to-face psychological interventions. Therefore, because of their safety, convenience, timeliness, and efficiency, online psychological counseling services have been widely established to provide free 24 h service on all days of the week for those in need (Liu et al., 2020; Zhao and Fan, 2020). The state and various social institutions provide a wealth of psychological service resources, but we do not know the degree to which college students use these resources. Previous studies have found that when individuals encounter psychological problems, they show a tendency to care for themselves first and then for others (Jiang and Xia, 2006). Professional psychological counseling or mental health services are not fully utilized by college students (Liang et al., 2017).

The phases-decision-making model (PDM) proposes that individual help-seeking behavior follows a three-stage internal decision-making process: stage 1 involves the perception of psychological problems; stage 2 involves self-service assessment, that is, the assessment of whether an individual has the willingness and ability to deal with the problems independently; and stage 3 involves other-assisted assessment, and turning to a professional institution for help is one of the options that individuals can consider. Possible solutions exist in each stage of the help-seeking process, and turning to professionals is considered at the end of the third stage. College students with poor mental health will turn to professional institutions and personnel for help only when all previous solutions are ineffective, showing a negative attitude toward help-seeking (Zhang et al., 2015). Research has suggested that convenience, economy, recipient self-efficacy, perception of the nature and severity of the problem, social tolerance of problem behavior, help-seeking behavior, previous help-seeking experience, and other determinants may be the main factors affecting the decision-making process, but there is a lack of relevant research evidence, and a large number of hypotheses still need to be tested (Jiang and Xia, 2006). However, during the COVID-19 epidemic, what is the utilization rate of mental health services among college students? What are the differences in mental health status between college students who use these service resources for psychological help and those who do not? What are the influencing factors? The answers are unknown and need to be studied. Therefore, by comparing the degree of psychological distress between college students who did and did not engage in psychological help-seeking, this study intends to determine the factors influencing college students’ help-seeking behavior.

## Materials and Methods

### Participants and Procedure

At the peak of the outbreak, we assessed students from 85 different universities in Guangdong Province using a brief self-administered online questionnaire that included demographic information, the COVID-19 Fear Screening Scale (CV-19FSS), the Patient Health Questionnaire (PHQ-9), and the Impact of Event Scale-6 (IES-6). We had prepared a normative notice applicable to these 85 schools, including the purpose, significance, deadline, and mode of participation of the survey. A contact person from each college was responsible for sending the above notice to each student via WeChat or QQ. Participants could use WeChat to access the survey and answer the online questionnaire anonymously by scanning the two-dimensional barcode or clicking on the relevant link from February 13 to February 22, 2020. An online consent form would be displayed on the front page of the online questionnaire; if participants had no objection to the objectives of the survey, they could officially start the survey by clicking the “next” button below, or they had the right to withdraw from the survey by closing the survey homepage. Each participant was only allowed to answer the questionnaire once. The whole process was entirely voluntary and non-commercial. In addition, all researchers involved in the survey had signed confidentiality agreements. Two sub-samples were formed according to the answer to the question “Have you ever sought psychological assistance in response to the COVID-19 epidemic situation?” and matched for age, sex, and grade. Participants who answered “yes” were defined as the counseling group, while those who answered “no” were defined as the non-counseling group. The study was approved by the appropriate institutional research and ethics committee.

### Measures

#### Demographics and Medical/Counseling Experience

Participants provided demographic information including age, gender, education level, psychiatric history (yes or no), and current location (i.e., Guangdong Province, Hubei Province, and other provinces). Basic information about their perceived mental health (good, general, or poor), experience with seeking psychological help (yes or no), and cognition of the local COVID-19 epidemic situation (peak, growth, flattening, turnaround, or uncertain) (assessed with items such as “What is your mental state at present?,” “Have you received psychological counseling services from professionals (counselors, psychiatrists, etc.) in the past?,” and “What is the current prevalence of COVID-19 in your region?”) was also collected.

#### Fear Related to COVID-19

The CV-19FSS is a 12-item self-report scale adapted from the SARS Fear Emotion Screening Inventory (Gao and Xie, 2005). The CV-19FSS is designed to assess the fear emotion during a public health emergency. All of the items are answered either “Yes” (1) or “No” (0), and the scores are subsequently summed to derive a total score for the scale. The scores range from 0 to 12, with higher scores indicating higher levels of fear. The CV-19FSS includes fear categories based on score ranges: scores of 10–12 indicate severe fear, scores of 7–9 indicate moderately severe fear, scores of 4–6 indicate mild fear, and scores of 0–4 indicate no fear. The Cronbach’s alpha for the CV-19FSS in the current sample was 0.799.

#### Level of Depressive Symptoms

The PHQ-9 is a nine-item self-report measure reflecting the diagnostic criteria for major depressive disorder. The Chinese version used in the current study was developed by Zhang et al. (2013). Participants were asked to rate how often each symptom bothered them during the past 2 weeks on a scale from 0 (not at all) to 3 (nearly every day). Total scores range from 0 to 27, with higher scores indicating a greater severity of depression. Scores of ≥ 5, ≥ 10, ≥ 15, and ≥ 20 represent mild, moderate, moderately severe, and severe depression, respectively (Martin et al., 2006). The PHQ-9 is a reliable, valid measure of depressive symptoms in the general population (Kocalevent et al., 2013). The Cronbach’s alpha for the PHQ-9 in the current sample was 0.816.

#### Level of Posttraumatic Stress Symptoms

The IES-6 is a useful screening instrument for epidemiological research and clinical practice. It was simplified by Thoresen on the basis of the revised version of the Impact of Events Scale (IES-R) and is strongly correlated with the IES-R (Thoresen et al., 2010). The IES-6 is a six-item self-report measure of psychological responses to trauma. Each item is rated on a Likert scale from 0 to 4, and its three subscales (Intrusion, Avoidance, and Hyperarousal) are closely affiliated with post-traumatic stress disorder (PTSD) symptoms. It can be anchored to any specific event, such as the COVID-19 outbreak. Clinically, the average scores of the IES-6 (the sum of the six items/6) are divided as follows: < 1.09, normal; ≥ 1.09 and < 1.5, PTSD is detected; and ≥ 1.5, may diagnose with PTSD (Asukai et al., 2002). The Cronbach’s alpha for the IES-6 in the current sample was 0.920.

#### Statistical Analysis

Data were analyzed with SPSS Version 22.0. Descriptive statistics, including frequencies and central tendencies, were calculated to characterize the sample’s demographic profile, fear level, depressive symptoms, and level of psychological trauma. A reliability test was used to check the internal consistency of the CV-19FSS, PHQ-9, and IES-6. The normal distribution of the quantitative data was checked using a one-sample Kolmogorov–Smirnov test. The results showed that the scores of fear, depression, and trauma were non-normal continuous variables. Differences between groups were tested via the Mann–Whitney U-test and Kruskal–Wallis H test for non-normal continuous variables and the chi-squared test or Fisher’s exact test for categorical variables whenever appropriate. Spearman’s correlation analysis was used to explore the relationships among fear, depression, trauma, psychiatric history, experience with seeking psychological help, and self-perceived mental health. Binary logistic regression analysis was performed to explore the potential factors influencing psychological help-seeking (counseling or non-counseling). Odds ratios (ORs) and 95% confidence intervals (95% CIs) were obtained from the logistic regression models. *P*-values of less than 0.05 were considered statistically significant (two-sided tests).

## Results

### Sociodemographic Profile

Based on data provided by the Guangdong Mental Health Committee of Colleges and Universities, a total of 361,969 college students completed the online survey, and 38,480 were excluded because they indiscriminately filled in information and selected the same option for each item. After for matching sex, age, and grade, we had a final sample of 4,164 participants, 2,082 of whom were in the counseling group and 2,082 of whom were in the non-counseling group. The proportion of respondents seeking psychological help in response to the COVID-19 epidemic was 0.64%.

The final sample included 2,164 (52.0%) males and 2,000 (48.0%) females, with 3,476 (83.5%) between 19 and 22 years old; 438 (10.5%) younger than or equal to 18 years old; and 250 (6.0%) between 23 and 25 years old. Half of them (*n* = 2,044, 49.1%) were freshmen, 1,262 (30.3%) were sophomores, 660 (15.9%) were juniors, and 198 (4.8%) were seniors. The majority were located in Guangdong Province (*n* = 3,874, 93.0%) at the time of the survey, while a small number were located in Hubei (*n* = 23, 0.6%) or other provinces (*n* = 267, 6.4%).

The comparison between the counseling group and non-counseling group is shown in Table 1. Participants who were living in an area where the COVID-19 epidemic was in a growth or peak period at the time of the survey had a significantly higher chance of being in the counseling group (χ^2^ = 22.372, d.f. = 4, *P* < 0.001). Those perceiving poor mental health, who had experienced mental illness, or who had sought psychological help had a significantly higher chance of being in the counseling group (χ^2^ = 151.647, d.f. = 2, *P* < 0.001; χ^2^ = 52.993, d.f. = 1, *P* < 0.001; χ^2^ = 269.295, d.f. = 1, *P* < 0.001). The number of students who could be diagnosed with PTSD in the counseling group was much higher than that in the non-counseling group (χ^2^ = 423.795, d.f. = 2, *P* < 0.001). The counseling group had a significantly higher likelihood of experiencing fear and depressive emotions (χ^2^ = 585.664, d.f. = 3, *P* < 0.001; χ^2^ = 259.218, d.f. = 4, *P* < 0.001).

**TABLE 1:** Comparison between subject groups (*n* = 4, 164).

Variable	All participants (%)	Counseling group	Non-counseling group	χ^2^	d.f.	*P*
**Current location**						
Guangdong Province	3,874(93.0)	1,931	1,943	0.732	2	0.694
Hubei province	23 (0.6)	13	10			
Other provinces	267 (6.4)	138	129			
**Local epidemic situation of COVID-19**						
Peak period	166 (4.0)	106	60	22.372	4	<0.001
Growth period	373 (9.0)	212	161			
Flattening period	1,486(35.7)	723	763			
Turnaround period	1,254(30.1)	613	641			
Uncertain	885 (21.3)	428	457			
**Perceived mental health**						
Good	3,640(87.4)	1,693	1,947	151.647	2	<0.001
General	434 (10.4)	308	126			
Poor	90 (2.2)	81	9			
**Psychiatric history**						
Yes	109 (2.6)	92	17	52.993	1	<0.001
No	4,055(97.4)	1,990	2,065			
**Experience with seeking of psychological help**						
Yes	457 (11.0)	394	63	269.295	1	<0.001
No	3,707(89.0)	1,688	2,019			
**Trauma level**						
Normal	1,621(38.9)	522	1,099	423.795	2	<0.001
PTSD detected	721 (17.3)	336	385			
PTSD diagnosed	1,822(43.8)	1,224	598			
**Fear degree**						
Not at all	1,817(43.6)	554	1,263	585.664	3	<0.001
A little	1,405(33.7)	810	595			
Too much	665 (16.0)	478	187			
Extreme	277 (6.7)	240	37			
**Depression**						
Not at all	2,479(59.5)	1,010	1,469	259.218	4	<0.001
Mild	1,131(27.2)	662	469			
Moderate	314 (7.5)	211	103			
Moderately severe	156 (3.7)	128	28			
Severe	84 (2.0)	71	13			

*COVID-19, coronavirus disease 2019. PTSD, post-traumatic stress disorder.*

### Group Differences in Mental Health State

The comparison of fear, depression, and trauma levels between the counseling group and non-counseling group is shown in Table 2. Levels of fear, depression, trauma, avoidance, intrusion, and hyperarousal were significantly higher in the counseling group (Z = −24.734, *p* < 0.001; Z = −16.541, *p* < 0.001; Z = −21.583, *p* < 0.001; Z = −16.984, *p* < 0.001; Z = −17.420, *p* < 0.001; Z = −19.582, *p* < 0.001). The results revealed that the counseling group had a worse mental health state.

**TABLE 2:** Comparison of fear, depression, and trauma levels (*n* = 4, 164).

Variables	Counseling group (*M ± SD*)	Non-counseling group (*M ± SD*)	*Z*	*P*^a^
Fear	5.50 ± 2.92	3.33 ± 2.36	–24.734	<0.001
Depression	5.92 ± 5.75	3.24 ± 4.06	–16.541	<0.001
Trauma	9.82 ± 4.69	6.84 ± 3.91	–21.583	<0.001
Avoidance	2.55 ± 1.97	1.56 ± 1.63	–16.984	<0.001
Intrusion	3.70 ± 1.96	2.68 ± 1.69	–17.420	<0.001
Hyperarousal	3.57 ± 1.65	2.60 ± 1.42	–19.582	<0.001

**M*, mean; *SD*, standard deviation. Avoidance, intrusion, and hyperarousal are subscales of the Impact of Event Scale-6 (IES-6). Fear, total score on the COVID-19 Fear Screening Scale (CV-19FSS); Depression, total score on the Patient Health Questionnaire (PHQ-9); Trauma, total score on the IES-6. *P*^*a*^ values were derived from the Mann–Whitney U-test.*

### Correlations Between the Studied Variables

As presented in Table 3, Spearman’s correlation analysis was used to explore the relationships among mental health state (trauma, fear, and depression) and demographic variables (perceived mental health, experience with seeking psychological help, and psychiatric history). Fear, depression, trauma, perceived mental health, and experience with seeking psychological help were significantly positively correlated with each other (*P*_s_ < 0.01). These results suggested that participants who had experience with psychological help-seeking and perceived poor mental health status had higher levels of fear, depression, and trauma. However, psychiatric history was positively correlated with depression, trauma, perceived mental health, and experience with psychological help-seeking (*P*s < 0.01) but not with fear (*P* = 0.146). This result showed that participants with a history of mental illness often turned to counseling, perceived worse mental health status, and had higher levels of depression and trauma.

**TABLE 3:** Correlations between mental health state and demographic variables.

Variables	1	2	3	4	5	6
1 Fear	1					
2 Depression	0.377**	1				
3 Trauma	0.516**	0.422**	1			
4 Perceived mental health	0.177**	0.362**	0.180**	1		
5 Experience with seeking psychological help	0.066**	0.150**	0.100**	0.165**	1	
6 Psychiatric history	0.023	0.132**	0.048**	0.160**	0.376**	1

Fear, total score on the CV-19FSS; Depression, total score on the PHQ-9; Trauma, total score on the IES-6; Perceived mental health: good, 1; general, 2; poor, 3; experience with seeking psychological help: yes, 1; no, 0; psychiatric history: yes, 1; no, 0. **p < 0.01.

### Associations Between the Studied Variables

The forward likelihood ratio test was used to screen the demographic variables (experience with seeking psychological help, perceived mental health, local epidemic situation, and psychiatric history) and mental health variables (depression, trauma, and fear) that had an influence on psychological help-seeking behavior by logistic regression. The categorical variables were transformed into dummy variables for analysis. The results showed that the regression model after excluding the two variables of psychiatric history and local epidemic situation had a good fitting effect (χ^2^ = 1,061.66, *P* < 0.01), and the prediction accuracy was 71.0%. Table 4 displays how psychological help-seeking behaviors are associated with mental health status (fear, depression, and trauma), experience of psychological help-seeking, and perceived mental health based on binary logistic regressive analysis. Mental health variables (fear, depression, trauma), experience with seeking psychological help, and perceived mental health can effectively predict college students’ psychological help-seeking behavior during the COVID-19 epidemic. When the scores of fear, depression, and trauma increased by one unit, the probability of college students seeking psychological counseling increased by 27% (95%CI = 1.23–1.31, *p* < 0.001), 2% (95%CI = 1.00–1.04, *p* = 0.032), and 8% (95%CI = 1.06–1.11, *p* < 0.001), respectively. During the COVID-19 outbreak, the probability of seeking psychological counseling was 7.06 times (95%CI = 5.27–9.45, *p* < 0.001) higher for college students with psychological help-seeking experience than for those without experience. The probability of seeking psychological counseling was 1.56 times (95% CI = 1.21–2.02, *p* = 0.001) and 3.61 times (95% CI = 1.68–7.76, *p* = 0.001) higher for college students who perceived general and poor mental health, respectively, than for college students with good perceived mental health.

**TABLE 4:** Binary logistic regression of whether college students seek psychological help or not.

Variables	B	S.E.	Wald	OR (95% CI)	*p*
Fear	0.24	0.02	230.01	1.27 (1.23–1.31)	< 0.001
Depression	0.02	0.01	4.59	1.02 (1.00–1.04)	0.032
Trauma	0.08	0.01	68.06	1.08 (1.06–1.11)	< 0.001
Experience with seeking psychological help	1.95	0.15	172.67	7.06 (5.27–9.45)	< 0.001
Perceived mental health					
Good				1 (reference)	
General	0.45	0.13	11.54	1.56 (1.21–2.02)	0.001
Poor	1.29	0.39	10.84	3.61 (1.68–7.76)	0.001
Constant	−2.01	0.09	527.00	0.13	< 0.001

*OR, odds ratio; 95% CI, 95% confidence interval.*

## Discussion

As is generally known, COVID-19 is highly infectious, spreads rapidly, and poses a challenge and threat to global public health security (Hiroshi et al., 2020). The Chinese Ministry of Education attaches great importance to the mental health of college students during the COVID-19 epidemic and has set up a number of psychological assistance hotlines. However, to date, the studies on this topic have been limited, and few have explored the psychological help-seeking and mental health status of Chinese college students during the COVID-19 outbreak. This study helped fill the research gaps described above, and it is the first large-scale survey to compare the mental health status of Chinese college students who sought and did not seek psychological counseling during the COVID-19 epidemic.

Our study highlighted a few major findings. First, during the COVID-19 outbreak, although college students faced many stressors, the rate of seeking psychological help was still low. Compared with those in the non-counseling group, college students who had sought psychological help experienced fear, trauma, and depressive symptoms more frequently. Second, the scores of fear, depression, and trauma during the COVID-19 epidemic can effectively predict the psychological help-seeking behavior of college students, and fear is the best predictor among them. Third, the experience with seeking psychological help and self-perceived mental health are also key variables for predicting the psychological help-seeking behavior of college students. College students who have experience with seeking psychological help and who perceive their mental health status as average or poor are more likely to seek psychological counseling during the COVID-19 outbreak.

Although the Ministry of Education and various social institutions have provided rich psychological service resources, the psychological help-seeking rate (0.64%) of college students in this study was still low, and a certain proportion of college students in the non-counseling group suffered from psychological symptoms but did not seek psychological help. This finding confirmed that college students are indeed a risk group for underutilization of mental health services, and they often hold a relatively negative attitude toward psychological counseling, consistent with previous studies (Jiang and Wang, 2003; Hunt and Eisenberg, 2010; Liang et al., 2017). In the face of psychological distress, college students often adopt informal ways to cope by seeking help from friends and family and seldom turn to professional psychological resources (Zhang et al., 2014). Many reasons have been proposed to explain why they do not seek professional help for common psychological distress. These include psychological factors such as negative attitudes toward seeking help, stigma, coping style, self-efficacy, personality, avoidance, passivity, worry about the evaluation of others, lack of understanding, unrealistic expectations of psychological counseling, and practical factors such as cost, transportation, or inconvenience (Zhou et al., 2010; Gulliver et al., 2010; Tan, 2012).

Psychological counseling was regarded as an imperative or last-resort choice by college students with poor mental health, and it was the final “exit” strategy adopted only when other channels for seeking help were blocked (Zhang et al., 2015). Seeking psychological help is an effective coping strategy, whereas not resorting to professional counseling even if suffering from serious mental issues is an evasive coping strategy. This type of help-seeking or coping strategy is consistent with the characteristics of collectivist cultures (including traditional Chinese culture). Avoidant coping, one of the major coping strategies in traditional Chinese culture, is positively related to Chinese young adults’ psychological symptoms (Tao et al., 2000) and is generally associated with greater psychological distress (Compas et al., 2001). However, Gan et al. (2004) found that avoidant coping may be more adaptive than active coping when facing uncontrollable stressors such as SARS-related stressors because when individuals engage in avoidant coping, they tend to ignore or avoid the source of stress and thus leave the situation unchanged so as to reduce the emotional stress elicited by a problematic situation. Moreover, stigma can also prevent college students from seeking psychological help. Many college students who are plagued by mental illness try their best to hide their illness when the explicit symptoms are not obvious, fearing that they will be labeled with a stigma once they ask for psychological help. Studies have shown that mental illness stigma (Fang, 2015) and self-stigma (Zhang and Hao, 2019) can lead to negative help-seeking attitudes, interfere with individuals’ choice of health-oriented actions, and hinder psychological help-seeking behavior.

According to the health belief model, the perception of disease susceptibility and severity is the core belief of behavior change, which depends on the individual’s understanding and evaluation of his/her own psychological problems as well as on the interpretation of the meaning of psychological symptoms. Psychological problems and emotional troubles are often expressed in the form of symptoms. A psychological symptom is a type of abnormal feeling state, and it is also the main manifestation of mental illness. When an individual regards his/her psychological problems as a manifestation of mental illness, he/she may have a positive attitude toward psychological help; otherwise, he/she may ignore the problem or try to solve it on his/her own. After all, most symptoms are transient and mild and do not constitute a diagnosis of the disease, and only meaningful symptoms can lead to health-oriented actions. Those who have suffered psychological pain but avoid counseling may, to some extent, define the psychological pain suffered during the COVID-19 epidemic as temporary, static, and a non-disease that can be alleviated with the control of the epidemic. This low perceived need would prompt them toward self-regulation rather than seeking psychological help (Jorm, 2012). On the other hand, the finding that the college students in the counseling group scored higher in fear, trauma, and depression could also be explained by the health belief model. Generally speaking, the stronger and more persistent the psychological problems or painful symptoms are and the greater the impact on the individual’s study, work, and life is, the easier it is to attract the attention of the parties concerned, which may lead to psychological help-seeking behavior (Liang et al., 2002). This is because when psychological distress is identified as a symptom, the perception of susceptibility and severity becomes clearer, which increases the likelihood of behavioral change (i.e., seeking professional psychological help).

The results of correlation and logistic regression analysis in the present study indicated that fear, depression, and trauma were all predictive factors of college students’ psychological help-seeking behaviors and that fear was the best predictor among them. This is in line with results from previous research that concluded that individuals who experienced more psychological symptoms during the epidemic were more actively seeking social support than were those with fewer symptoms (Alexandra et al., 2011). With the extremely high infection rate and relatively high mortality rate, individuals, families, and communities experience feelings of hopelessness, despair, grief, bereavement, and a profound loss of purpose due to the COVID-19 pandemic (Levin, 2019). Feelings of loss of control drive fear and uncertainty as the trajectory of the pandemic constantly evolves (Usher et al., 2020). Many studies have suggested that the COVID-19 outbreak has already unleashed and exacerbated fear (Ahorsu et al., 2020; Ren et al., 2020). Fear has been conceptualized as a causal factor in mental distress (Lester, 2003), in severe cases resulting in PTSD and/or depression (Perrin et al., 2009).

However, fear is not only a common known response to infectious disease outbreaks but also a stress response to public health emergencies. Based upon transactional theory (Lazarus and Folkman, 1984), stress is described as an interactive process between stressors and an individual’s psychological responses (e.g., appraisal, coping, adjustment). When confronted with a stressful situation, the body initiates the “fight or flight response,” and over time, the body may become exhausted, leading to physical and psychological burnout (Melamed et al., 2006). Several studies have pointed out many problems caused by fear during infectious outbreaks, including accelerated disease transmission (Shultz et al., 2016), economic downturn (Lempel et al., 2009), a decline in immune function (Segerstrom et al., 1998) and mental health (Silver et al., 2013), and delays in making help-seeking decisions (Ren et al., 2020). Although fear causes many problems, it also motivates individuals to seek help and cope and thereby drives the contemporary mental health system (Lester, 2003). To deal with stress at its root and restore psychological energy, individuals need to try to do positive things to reduce stress and engage in active coping. Calling a psychological hotline is a primary way for individuals to solve psychological problems during the COVID-19 epidemic. Using such a hotline can help individuals by increasing their psychological motivation, enhancing their psychological strength, stimulating their initiative and autonomy, building their confidence in their ability to overcome their own problems, and helping them to gain a sense of control and certainty (Duan, 2007). For college students who were tortured by fear during the COVID-19 epidemic and were seeking ways to avoid being overwhelmed by the psychological exhaustion caused by the accumulation of pressure and problems, turning to psychological counseling was a concrete manifestation of active coping.

We also found that self-perceived mental health and experience with seeking psychological help play important roles in predicting the psychological help-seeking behaviors of college students. This is in accordance with the results from previous studies (Jiang and Xia, 2006; Lu, 2018). A 6-month follow-up survey of 216 college students facing psychological problems conducted by Li et al. (2016) found that the level of perception of psychological problems can positively predict the level of seeking professional help. The self-rating of perceived mental health has been shown to be stable from ages 23–33 and to be related to psychological distress (Mano et al., 2001). The health belief model and the PDM jointly emphasize the importance of individuals’ perception and judgment of their psychological problems, which is often the first step in the help-seeking process. As an extension of health belief theory, protection motivation theory emphasizes the moderating effect of cognitive processes between attitude and behavioral changes, including threat assessment and coping evaluation, which together form protective motivation and then promote the occurrence or maintenance of behavior. The self-perception of mental health is one of the important factors in the process of threat assessment. The perception of poor mental health status by an individual will initiate threat assessment and promote protective motivation, achieving behavioral change (i.e., seeking psychological help).

In terms of psychological help-seeking experience, those who have sought psychological help showed a more positive attitude and a stronger desire for help from psychologists. This is mainly related to these individuals’ sense of self-efficacy and the expected benefits of psychological counseling. According to previous research in the field of professional psychological help-seeking, efficacy is mainly divided into two categories: individual self-efficacy in dealing with psychological distress and self-efficacy as a client of psychological counseling. Zhao (2008) reported that the lower an individual’s self-efficacy is for dealing with psychological problems, the higher the willingness to seek help, because the person feels that it is necessary to turn to others to solve the problem. On the other hand, self-efficacy as a client of psychological counseling is positively correlated with professional psychological help-seeking attitudes. If individuals think that they can benefit from counseling, then their help-seeking attitude will be more positive (Liu, 2012), and their willingness to seek help will be stronger (Wang and Sun, 2008); this consequently results in a higher likelihood of choosing to seek psychological counseling (Xia and Jiang, 2007). In addition, it is typical to consider the expected benefits (the possible benefits of asking for help) before making a decision to ask for help. From the perspective of motivation theory, when an actor engages in a behavior, the target object of the behavior should be an inducement to the actor, and to a certain extent, the target object should be able to meet the needs of the actor and bring benefits to the actor. It is impossible for actors to pursue goals that are disadvantageous to them (Zhang, 1999). Therefore, it is not difficult to understand that a good psychological help-seeking experience can increase college students’ sense of self-efficacy and fulfill their need to resolve their psychological problems, which increases their probability of seeking psychological counseling in the future.

## Limitations

The study has several limitations. First, the cross-sectional design could not explain the cause–effect relationships and does not allow the investigation of changes in individuals’ mental health status and psychological help-seeking behavior across different periods of the COVID-19 epidemic, which could more fully reflect the psychological status and psychological help-seeking behavior characteristics of college students during the outbreak. Second, because all the constructs were assessed by self-report, the estimated relations among fear, depression, trauma, and psychological help-seeking behaviors might be subject to response bias. Future research should adopt multi-informant and multimethod assessment approaches. Third, due to the use of the convenience sampling method, the study sample primarily comprised college students in Guangdong Province. Future research needs to expand the scope of the survey to other provinces and cities in China and carry out stratified sampling to obtain a more comprehensive understanding of the situation of Chinese college students. Fourth, this survey lacked the measurement of the three processes of seeking psychological help, namely, help-seeking attitudes, intentions, and behaviors, limiting the explanatory power of the results. Future research can supplement the measurement of these key indicators, to better show the change in the psychological help-seeking process in the context of the epidemic.

## Conclusion

To summarize, in the context of public health emergencies, the rate of seeking psychological help was still low, and college students with a poor psychological condition turn more to seeking psychological counseling. Fear caused by the COVID-19 outbreak is more likely to predict college students’ psychological help-seeking behavior than depression and trauma because it not only causes a stress response but also strengthens college students’ motivation to seek help. In addition, college students with good psychological help-seeking experience and poor mental health status have a higher probability of seeking psychological help, which may be related to self-efficacy and expected benefits.

As one of the few studies on mental health and psychological help-seeking behavior among college students during the COVID-19 epidemic, this study has important implications for university counseling services with respect to preventing, identifying, and treating mental health problems among students during acute, large-scale stressors such as an infectious disease outbreak. As students who were not directly affected by COVID-19 reported significant numbers of COVID-19-related psychological symptoms during the epidemic, university campuses should develop and implement effective screening procedures to closely monitor students’ exposure to stressors and mental health status. Moreover, fear is the key factor motivating college students to seek psychological help. We should design a psychological intervention program for fear and fully utilize psychological assistance hotlines to help college students better adjust themselves. Last but not least, performing psychological help-seeking intervention, strengthening the dissemination of mental health knowledge, and improving the level of mental health perception are effective ways to improve help-seeing attitudes and increase the probability that college students will seek psychological help.

## Data Availability Statement

The data analyzed in this study is subject to the following licenses/restrictions: The data is related to the subjects’ personal privacy. Requests to access these datasets should be directed to S-WL, 357772263@qq.com.

## Ethics Statement

The studies involving human participants were reviewed and approved by the Institutional Ethics Board of Southern Medical University. Written informed consent for participation was not required for this study in accordance with the national legislation and the institutional requirements.

## Author Contributions

S-WL, R-NC, X-GL, J-BC, and S-YT sorted out the data. S-WL and R-NC did the literature search and analyzed the data. S-WL wrote the manuscript. L-LL provided the statistical methods to improve the manuscript. J-BZ directed the study design, revised the manuscript, and modified the language. All authors contributed to the article and approved the submitted version.

## Conflict of Interest

The authors declare that the research was conducted in the absence of any commercial or financial relationships that could be construed as a potential conflict of interest.
